# Role of innate immunological/inflammatory pathways in myelodysplastic syndromes and AML: a narrative review

**DOI:** 10.1186/s40164-023-00422-1

**Published:** 2023-07-08

**Authors:** Charan Thej Reddy Vegivinti, Praneeth Reddy Keesari, Sindhusha Veeraballi, Catarina Maria Pina Martins Maia, Ansh Krishnachandra Mehta, Rohit Reddy Lavu, Rahul Kumar Thakur, Sri Harsha Tella, Riya Patel, Venkata Kiranmayi Kakumani, Yashwitha Sai Pulakurthi, Srinivas Aluri, Ritesh Kumar Aggarwal, Nandini Ramachandra, Rongbao Zhao, Srabani Sahu, Aditi Shastri, Amit Verma

**Affiliations:** 1 Department of Medicine, Jacobi Medical Center/Albert Einstein College of Medicine, Bronx, NY 10461 US; 2 Department of Oncology, Albert Einstein College of Medicine, Bronx, NY 10461 US; 3 Department of Medicine, Staten Island University Hospital, Staten Island, NY 10305 US; 4 Department of Medicine, Saint Michael’s Medical Center, Newark, NJ 07102 US; 5 Department of Hematology and Oncology, National Institute of Health, Bethesda, MD 20892 US; 6 Department of Hematology and Oncology, Jacobi Medical Center/ Albert Einstein College of Medicine, Bronx, NY 10461 US; 7 Department of Oncology, Yashoda hospitals, Hyderabad, 500036 India; 8 Department of Medical Oncology, Mayo Clinic, Rochester, MN 55905 US; 9 Department of Hematology and Oncology, University of Buffalo - Roswell Park Comprehensive Cancer Center, Buffalo, NY 14203 US; 10 Harvard Medical Faculty Physicians, Beth Israel Deaconess Medical Center, Boston, MA 02215 US; 11 Department of Oncology, Blood Cancer Institute, Montefiore Medical Center/Albert Einstein College of Medicine, Bronx, NY 10461 US

**Keywords:** Myelodysplastic syndromes (MDS), Acute myeloid leukemia (AML), Innate Immune pathways, Tumor microenvironment (TME), Toll like receptors (TLRs), IL1 receptor Associated kinase (IRAK), Inflammasome

## Abstract

Dysregulation of the innate immune system and inflammatory-related pathways has been implicated in hematopoietic defects in the bone marrow microenvironment and associated with aging, clonal hematopoiesis, myelodysplastic syndromes (MDS), and acute myeloid leukemia (AML). As the innate immune system and its pathway regulators have been implicated in the pathogenesis of MDS/AML, novel approaches targeting these pathways have shown promising results. Variability in expression of Toll like receptors (TLRs), abnormal levels of MyD88 and subsequent activation of NF-κβ, dysregulated IL1-receptor associated kinases (IRAK), alterations in TGF-β and SMAD signaling, high levels of S100A8/A9 have all been implicated in pathogenesis of MDS/AML. In this review we not only discuss the interplay of various innate immune pathways in MDS pathogenesis but also focus on potential therapeutic targets from recent clinical trials including the use of monoclonal antibodies and small molecule inhibitors against these pathways.

## Background

Myelodysplastic syndromes (MDS) are myeloid clonal disorders characterized by ineffective hematopoiesis and bone marrow dysplasia, and they are often associated with chronic inflammatory conditions and an increased risk of transformation to acute myeloid leukemia (AML) [1]. Multiple mechanisms operate in the pathogenesis of MDS, including genetic and epigenetic mutations and apoptotic and differentiation abnormalities, but there is increasing recognition of the role of the innate inflammatory microenvironment. Genetic abnormalities and aging can influence the pathogenesis of MDS via alterations in the inflammatory microenvironment [2]. Understanding the specific immune pathways in the pathogenesis of MDS and how they operate differently in low-risk and high-risk MDS patients is crucial to developing future therapeutics for MDS. This review elaborates on the various innate immune pathways involved in the pathogenesis of MDS and AML and the clinical implications of the same.

**Fig. 1:**
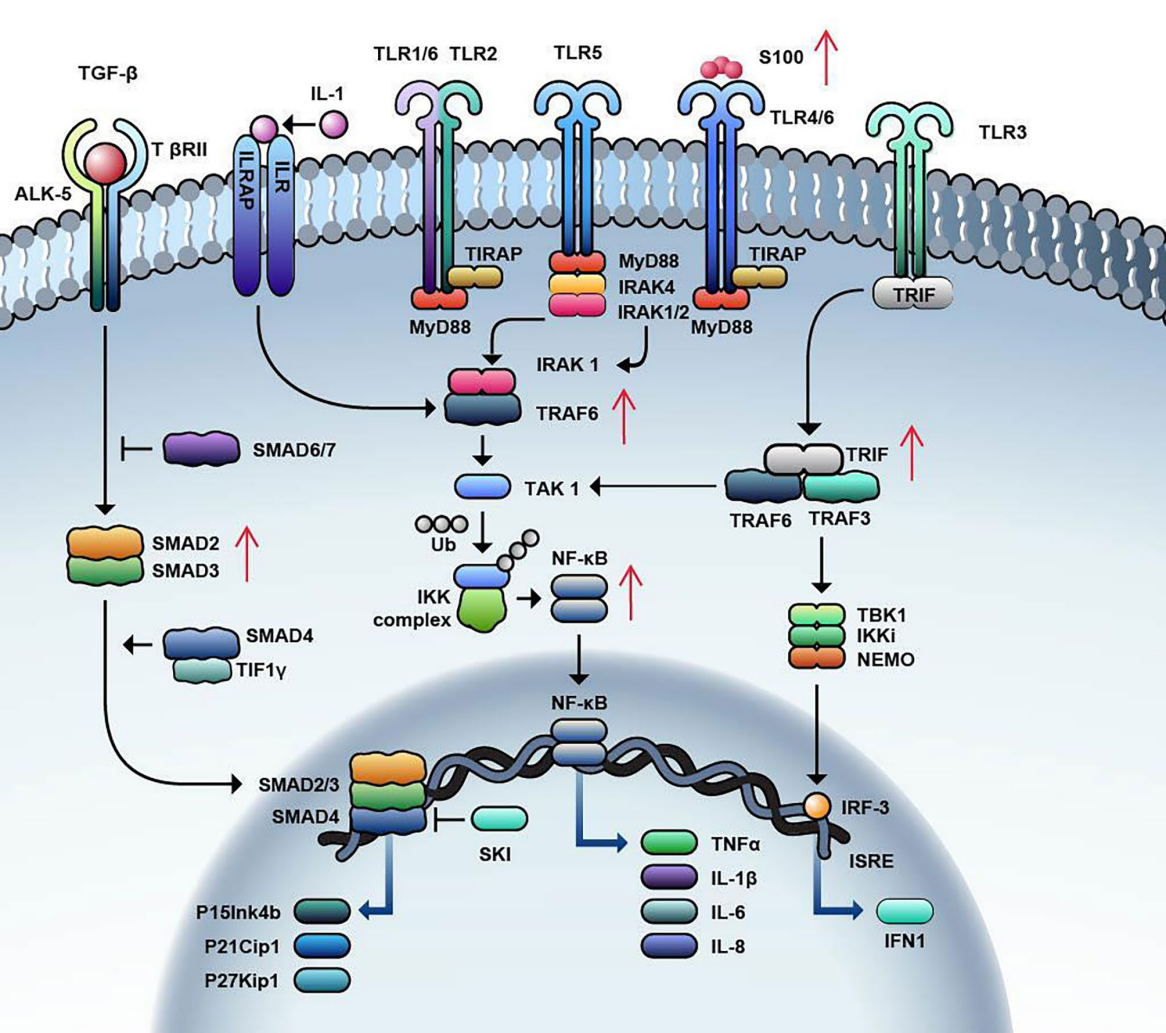
Innate Immune Pathways involved in MDS

Toll like receptors (TLRs) on binding to ligands, undergoes conformational change, and leads to recruitment of an adapter protein called MyD88 leading to activation of IRAK4 and IRAK1. Once activated, IRAK-1 binds to TRAF-6 which consequently activates TAK1, leading to phosphorylation of IKK complex and activation of transcription factor NF-κβ. Activation of NF-κβ can also occur through TRIF, not dependent on MyD88. Binding of TGF-β ligand to TGF-β receptor leads to activation on SMAD2/3, forms a complex with SMAD4 leading to upregulation of CDK inhibitors such as p15Ink4b, p21Cip1, and p27Kip1. In MDS, there is increased signalling and downstream molecules of TLR pathway which includes IRAK4, TRAF-6 and NF-κβ. TGF- β pathway is overactivated leading to increased levels of SMAD2 and SMAD3.

### Role of Toll like Receptors (TLRs) Signaling in MDS

TLRs are pattern recognition receptors (PRRs) and major components of the innate immune system. TLRs recognize pathogen-associated molecular patterns (PAMPs) and damage-associated molecular patterns (DAMPs) [2]. The drugs targeting TLR pathway have been discussed in Table 1.

**Table 1:** Potential therapeutic agents targeting the inflammatory pathways in myelodysplastic syndromes

Target Molecule	Drug	Phase	Type of MDS	Proposed mechanism	References
TLR2	Tomaralimab (OPN-305)	I/II	Low	IgG4 monoclonal antibody against TLR2	NCT02363491
TLR2	Bortezomib	II	Low	TRAF6 inhibitor via bortezomib-mediated autophagy	NCT01891968
IRAK4	Emavusertib (CA-4948)	II	High risk	IRAK4 inhibitor	NCT04278768
TGF-Beta	Luspatercept	III	Low	Binds to endogenous TGF-β superfamily ligands, thereby diminishing Smad2/3 signaling.	NCT02621070 (MEDALIST trial)
TGF-Beta	Galunisertib	II	Low	Inhibitor of the TGF-β receptor type 1 kinase (ALK5)	NCT02008318
Inflammasome	MCC950	Preclinical		Blocks the NACTH ATPase domain of NLRP3	Ashley A et al. [65]
Inflammasome	3,4 methylenedioxy-β-nitrostyrene (MNS)	Preclinical		Inhibits NLRP3 ATPase activity by cysteine modification	Chakraborty et al. [66]
Inflammasome	CY-09	Preclinical		Inhibits NLRP3 ATPase activity to block NLRP3 activation	Jiang Hua et al. [67]
Inflammasome	Ibrutinib	I	High	BTK inhibitor; directly binds ASC and NLRP3; inh ASC speck formation	NCT03359460
P38 MAPK	Pexmetinib (ARRY614)	I	Low or intermediate	Enhancing Megakaryopoiesis	NCT01496495
P38 MAPK	Talmapimod (SCIO-469)	II	Low	Enhancement of Hematopoiesis and reduction of apoptosis	NCT00113893
IL-8 receptor/CXCR2	SB-332,235	Preclinical		Reduction of growth and colony forming in MDS BM cells	Schinke C et al. [93]
IL-8	BMS-986,253	I/II		Anti-il-8 monoclonal antibody	NCT05148234
Caspase	CWP232291	1	High risk	Inhibitor of Wnt signaling that causes degradation of β-catenin via apoptosis	NCT01398462

#### Mechanism of signaling

TLRs have an ectodomain, a transmembrane domain, and a cytoplasmic domain. The ectodomain recognizes specific PAMPs or DAMPs followed by activation of MyD88 dependent or independent pathway. TLR1, TLR2, TLR4, and TLR6 recruit the Toll/interleukin-1 (IL-1) receptor (TIR) domain-containing adapter protein (TIRAP). The TIR domain of TIRAP binds the TIR domain of TLR2 and recruits MyD88. TL3 and TLR4 uses a TIR domain-containing adapter-inducing interferon-beta (IFN-beta) (TRIF) pathway, which is independent of MyD88 signaling [3].

In the MyD88 dependent pathway, MyD88 recruits members of the serine-threonine kinase interleukin-1 receptor-associated kinase (IRAK) family of proteins (IRAK4, IRAK1, and IRAK2), and forms a complex called Myddosome [4]. During complex formation, IRAK4 activates IRAK1, which is then autophosphorylated at multiple sites followed by the release of activated IRAK-1 from MyD88 [5]. The activated IRAK-1 binds to the E3 ubiquitin ligase tumor necrosis factor (TNF) R-associated factor 6 (TRAF6), which in turn activates the transforming growth factor beta-activated kinase 1 (TAK1). TAK1 binds to the IKK complex through ubiquitin chains, which results in phosphorylation and the activation of IKKβ [4]. This also results in the phosphorylation of the NF-kB inhibitory protein IκB⍺, which causes proteasome degradation, thereby permitting NF-κβ to translocate into the nucleus and induce proinflammatory gene expression [4].

In the TRIF dependent pathway, TRIF activation causes the recruitment of TRAF6 and TRAF3 [6]. TRAF-6 recruits the Receptor-interacting protein (RIP) -1 kinase, which results in the activation of the TAK-1 complex [6]. The activated TAK-1 complex causes the activation of the NF-kB and MAPKs pathways, thereby resulting in the production of inflammatory cytokines [6]. In contrast, TRAF3 recruits the TANK-binding Kinase 1 (TBK 1), IκB kinases (Ikki), and NEMO for the phosphorylation and dimerization of the transcription factor Interferon Regulatory Factor 3 (IRF-3) [6]. Subsequently, IRF3 nuclear translocation and inducement of the expression of type I interferon (IFN) by stimulating IFN stimulated response elements (ISREs) [6].

#### Role of TLRs in hematopoiesis

TLRs are widely expressed in hematopoietic cells such as dendritic cells, macrophages, lymphocytes, and non-hematopoietic cells like fibroblasts cells and epithelium [7]. TLRs are also expressed in early hematopoietic progenitors and stem cells [8]. In vitro studies demonstrated that when lineage marker negative, stem-cell antigen positive-1, c-kit positive bone marrow cells (Lin(-) c-Kit(+) Sca-1(+) – LKS cells) from wild type mice were transplanted into TLR2, TLR4, or MyD88 knockout mice and then exposed to Pam3CSK4 (a TLR2 agonist), LPS (a TLR4 agonist), or CpG oligodeoxynucleotide (a TLR9 agonist), the cells differentiated preferentially towards macrophages [9]. In vitro stimulation of hematopoietic progenitors with Pam3CSK4 (a TLR2 agonist) or LPS (a TLR4 agonist) also showed TLR signaling, which drives myeloid differentiation in a MyD88-dependent manner [8, 10]. Stimulation of TLR2 and TLR4 in mice leads to the production of one of the principal cytokines, GCSF, and results in the releasing of bone marrow HSPCs and myeloid precursors into the peripheral blood [11, 12]. All these studies demonstrate that TLRs have important roles in myeloid differentiation of hematopoetic stem cells.

#### TLRs in the pathogenesis of MDS

In MDS, there is an increased expression of TLR2 and its binding partners TLR4 and TLR6 in the bone marrow CD34 cells [13]. In lower-risk MDS patients, TLR2 expression was highest compared to high-risk MDS patients and healthy controls, and it correlated with a better overall survival. Whereas in higher-risk patients, TLR6 expression was highest [13–15]. In vitro studies demonstrated that TLR2 expression directly correlates with the apoptosis of CD34 cells, mostly occurring in the early stage of MDS due to the increased expression of pro-apoptotic molecules, such as Bax and Bad [16]. TLR2 induced CD34 + apoptosis is due to upregulation and nuclear translocation of beta arrestin 1, which is significantly elevated in patients with low-risk disease compared to those with higher-risk MDS or healthy controls [15]. In vitro studies demonstrated that the knockdown of β-arrestin1 in cultured CD34 + cells mitigated TLR2 agonist-induced cell death [15]. In addition, 11% of the MDS patients had a genetic variant of the TLR receptor, TLR2-F217S, which resulted in the robust activation of NF-κB upon TLR2 activation [13].

### Role of myeloid differentiation in primary response protein 88 (MYD88) signaling in MDS

MyD88 was originally identified as a myeloid differentiation primary response gene, which is upregulated during IL-6 induced macrophage differentiation [17]. MyD88 is an important component of the signaling pathway mediated by the IL-1 and IL-18 receptors, which are responsible for TH1 cell differentiation and Interferon γ production [18].

#### Role of MYD88 in hematopoiesis

MYD88 is the key mediator of Toll-like receptor signaling except for TLR3 [19, 20]. Studies on mice showed that MyD88 influences both myeloid and lymphoid cell development in the bone marrow, and that it is also associated with early and late hematopoiesis [21]. Bruton tyrosine kinase (BTK) is associated with MYD88 in B cells and is involved in B cell signaling in the development and functioning of adaptive immunity [22].

#### MYD88’s role in the pathogenesis of MDS

MYD88 mutations are commonly identified recurring mutations in chronic lymphocytic leukemia (CLL), B-cell lymphoma, and Waldenstrom’s macroglobulinemia [23–25]. MYD88 RNA levels are higher in MDS patients and is associated with shorter overall survival (OS) [26]. The blocking of homodimerization of MYD88 in the CD34 + cells of lower-risk MDS patients led to a 1.6 to 2-fold increase in erythroid and a 30% increase in the total number of colonies; this effect was not observed in high-risk MDS patients [26]. Erythroid differentiation that occurs after the MYD88 blockade is positively correlated with an increased ratio of *GATA1/GATA2* genes and the expression of CD71, EPOR, GYPA, and GYPB in CD34 + bone marrow cells [26].

### Role of NF-κB (nuclear factor kappa light chain enhancer of activated B cells) signaling in MDS

The NF-κB signaling pathway is involved in the production of inflammatory cytokines, chemokines, and adhesion molecules, and it also regulates apoptosis, cell proliferation and differentiation, and the activation of macrophages, granulocytes, osteoclasts, dendritic cells, and erythrocytes [27, 28]. NF-κB signaling pathway leads to myeloid differentiation by activating granulocyte macrophage colony stimulating factor (GMCSF), a cytokine that promotes differentiation of bone marrow stem cells towards granulocyte and monocytes [27, 29]. In addition, the NF-κB can also act as both anti-apoptotic or a pro-apoptotic regulatory factor based on cell type and stimuli [30].

#### NF-κB’s role in the pathogenesis of MDS

NF-κB activity is significantly elevated in MDS patients especially in those over the age of 75 [31]. In a study involving de novo MDS patients, the activation of NF-κB was significantly associated with increased ferritin (≥ 500 ng/mL), percentage of blasts (≥ 5%) and IL-8 levels [31]. NF-κB is activated in the mesenchymal cells of patients with low-risk myelodysplastic syndromes, resulting in the attenuation of the HSPCs function [32]. NF-κB signaling in human mesenchymal cells results in the upregulation of inflammatory markers such as IL6, IL8, CCL3, S100A9, INHBA, and CCL5 resulting in the impaired proliferation of mesenchymal cells, reducing support to HSPC, thereby attenuating the number and function of HSPCs, as reflected by reduced CFU-Colonies [32]. High-risk MDS and AML bone marrow samples express strong constitute activation of NF-κB. Inhibition of NF-κB activation results in increased apoptosis of MDS blasts cells, so NF-κB can be a potential therapeutic target in MDS [33].

### Role of toll-interleukin 1 receptor (TIR) domain-containing adapter protein (TIRAP)

TIRAP, also known as MyD88-adaptor Like (MAL), is a key intracellular adaptor molecule not only associated with the signaling of TLR2 and TLR4, but also involved in MYD88 independent inflammatory signaling [34]. The IFN-γ-TIRAP pathway is involved in bone marrow failure and MDS [35]. An analysis of the gene expression in the CD34 + cells of MDS showed that TIRAP expression was increased in del 5q MDS [35, 36]. Mice transplanted with TIRAP-expressing HSPCs showed an overall decreased survival rate due to bone marrow failure and significant pancytopenia [35]. The TIRAP-transduced bone marrow of transplanted mice reduced the number of viable common myeloid progenitors (CMP) and granulocyte–monocyte progenitors (GMP) resulting in pancytopenia [35]. The high mobility group box 1 (HMGB1) belongs to a non-histone protein localized in the nucleus, where it acts as a DNA chaperone to help in DNA repair and maintenance [37]. It can also be seen on an extracellular surface, where it functions as a DAMP. It plays a significant role in inflammatory diseases and cancers and acts as an alarmin in promoting AML progression [37, 38]. The overexpression of TIRAP in hematopoietic cells releases IFN-γ, which can act in two ways. IFN-γ can directly affect megakaryopoiesis and erythropoiesis in the bone marrow, and secondly, it can indirectly suppress myelopoiesis through the release of HMGB1 (alarmin), which disrupts the bone endothelium, resulting in bone marrow failure [35, 36]. In vivo experiments demonstrated that blocking HMGB1 in the presence of TIRAP expression resulted in the reversal of the bone marrow endothelial defect and the restoration of myelopoiesis [36].

### Role of TNF receptor-associated factor 6 (TRAF-6) in MDS

Tumor necrosis factor receptor-associated factor 6 (TRAF6) is essential for maintaining HSC quiescence and controlling myeloid-biased differentiation through minimal NF-κB signaling via cyclin dependent kinase inhibitors, which are negative regulators of cell cycle progression [39, 40]. Myeloid-derived suppressor cells (MDSCs) are premature heterogenous group of myeloid cells derived from bone marrow, and they have the ability to suppress the immune responses against a tumor [41]. The signal transducer and activator of transcription 3 (STAT3) is a transcription factor that is activated by growth factors and cytokines, and it plays an important role in MDSCs activation, function, and expansion [42, 43]. E3 ubiquitin ligase TRAF-6 binds to STAT3, leading to the phosphorylation of STAT3, which results in the activation of MDSCs [41]. TRAF-6 knockdown in mice attenuated MDSCs role in accelerating tumor progression as the inhibitory effect of MDSCs on CD4 + T cell proliferation was significantly decreased [41]. TRAF-6 expressing bone marrow transplanted mice progressed either to bone marrow failure or developed AML [44]. Activation of the canonical pathway, i.e., TLR4–TRAF6–NF-κB activation has been reported in MDS. The 5q deletion correlated with the loss of two miRNAs (miR-145 and miR-146a), which resulted in an increased expression of TRAF-6 in MDS cells [44]. The knocking down of miRNAs in mice models resulted in significant thrombocytosis, mild neutropenia, and megakaryocytic dysplasia [44]. IL-6 production and persistent elevation seen in TRAF-6 transplanted mice is responsible for platelet survival and production [44].

**Fig. 2:**
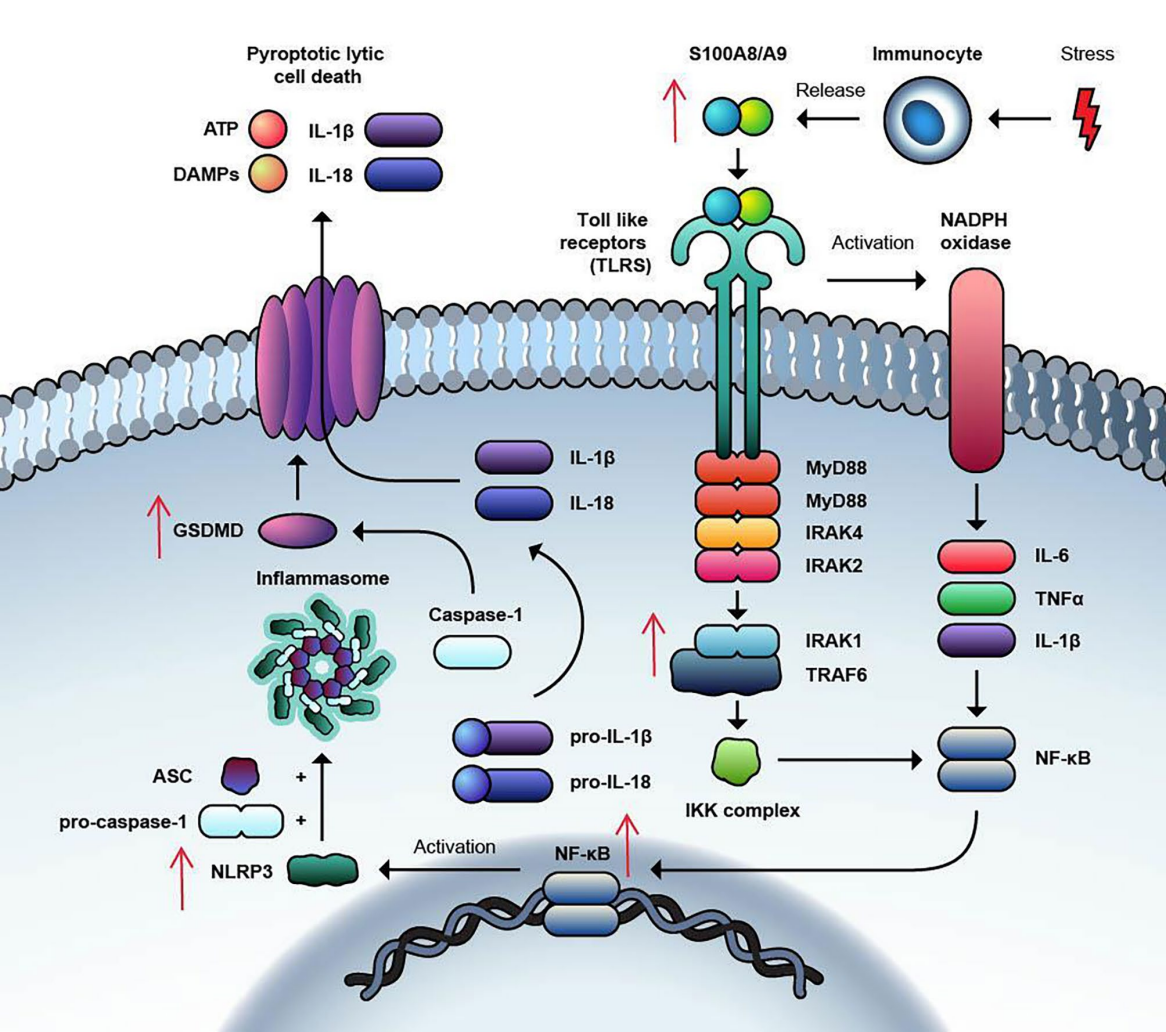
Inflammasome Pathway

#### Inflammasome pathway

S100A8/A9 released from immunocytes and MDSCs upon binding to TLRs/CD33/RAGE receptors not only activates NF-κB through IRAK1-TRAF6 but also activates the NADPH oxidase leading to generation of proinflammatory mediators like IL-6, TNF alpha, and IL-1 Beta which in turn stimulate NF-κB. NF-κB activation leads to downstream NLRP3 inflammasome assembly that leads to caspase-1-dependent pro-inflammatory cell death through the activation of inflammatory cytokines like interleukin 1β (IL-1β) and IL-18, and pore formation leading to osmotic lysis. In MDS, there is increased signalling of TLR pathway via S100 A8/A9 and inflammasome formation leading to cell death.

### Role of IL1 receptor associated kinases (IRAK) in MDS

IL1-receptor associated kinases (IRAK) are a family of intracellular serine threonine kinases that play a significant role as signal transduction mediators of TLR and interleukin–1 receptor signaling [45]. More recently, some groups have reported that IRAK 1 regulates the rapid NLRP3 inflammasome assembly and caspase 1 cleavage in a transcription independent fashion. NRLP3 assembly is dependent on the catalytic competency of both IRAK1 and IRAK 4 [46, 47] (Fig. 2).

#### IRAK1

IRAK1 mRNA is overexpressed in ~ 20–30% of MDS patients. More remarkably, the IRAK1 protein is overexpressed and in a hyperactivated state in the majority of the MDS marrow samples examined [48]. Studies with IRAK-Inh and IRAK1 knockdown in MDS cell lines/human MDS samples demonstrated dramatic impairment of MDS cell proliferation, progenitor function, and viability in vivo and in vitro. However, a subset of MDS/AML progenitors escape IRAK-Inh apoptosis and had persistence of anti-apoptotic BCL2 like proteins. It has been demonstrated that a combination of BCL2/IRAK inhibitors in this subset suppressed MDS clones, which can be a potential target to treat MDS [48]. IRAK1 is also negatively regulated by microRNAs (miR-145 and mir-146a), thereby increasing IRAK-1 levels and inflammation in MDS patients with del miR.

#### IRAK4

IRAK4 has two protein isoforms based on the inclusion and exclusion of exon-4 [49]. The inclusion of exon 4 in the mRNA resulted in a longer 460 residue IRAK4-L protein, which has a death domain, hinge domain, and kinase domain. The exclusion of exon-4 resulted in a shorter 336 residue IRAK-S, which has a hinge and kinase domain and lacks a death domain [50]. U2 small nuclear RNA auxiliary factor 1 (*U2AF1*) is a gene encoding an RNA-binding protein critical for recognition of AG dinucleotide in pre-mRNA 3´ splicing site [51]. The *U2AF1* gene is located on 21q22.3, and its mutations are seen in approximately 7–11% of MDS patients [52–54]. *U2AF1* S34 mutants promote alternate splicing resulting in the inclusion of exon 4 and expression of IRAK4-L isoform [55]. In the study conducted by Smith et al., 100% of MDS patients with *U2AF1* mutations expressed IRAK4-L, whereas 52% of MDS patients without splicing-factor mutations expressed IRAK4-L [50]. Mice xenografted with primary *U2AF1*-mutant cells showed a 50% reduction in MDS cells after 3 weeks of treatment with IRAK4 kinase inhibitor (CA-4948) [50].

*SF3B1* is the largest component of the SF3B complex that stabilizes the binding of U2 snRNP to the branch site during pre-mRNA splicing [56]. The *SF3B1* gene is located on chromosome 2q33. Mutations in this gene are observed in approximately 35–43% of MDS cases and 98% of myelodysplastic syndrome–ringed sideroblasts (MDS- RS) [57, 58]. RNA-Seq splicing analysis of *SF3B1* Mutant MDS samples showed retention of exon 6 of IRAK4, resulting in IRAK4-L, which contains the entire death domain leading to NF-kB activation and blocking hematopoietic differentiation [59]. In MDS cells with primary SF3B1 mutation, inhibiting IRAK4 with CA-4948 decreased NF-kB activation and production of inflammatory cytokines, along with an increase in myeloid colony, myeloid differentiation, and reduction in leukemic growth in a xenografted mice model [59].

AML cells have significantly higher expression of IRAK4-L, whereas normal bone marrow-derived CD34 + hematopoietic cells predominantly express IRAK4-S [50]. The N-terminal death domain of IRAK4-L interacts directly with MyD88, initiating myddosome formation, resulting in autophosphorylation of IRAK-4, and subsequently activating IRAK-1, which facilitates recruitment of TRAF6 and NF-B activation [60]. IRAK4-S lacks an N-terminal death domain and does not interact with MyD88 [50]. On treatment with ATP-competitive IRAK4 kinase inhibitor (CA-4948), IRAK4-L expressing MDSL, TF1, and THP1 cells generated fewer leukemic progenitor colonies, although IRAK4-S expressing HL60, F36P, and normal CD34 + cells were unaffected. Thus, inhibition of IRAK4 kinase activity in MDS or AML cell lines expressing IRAK4-L decreased leukemic function in vitro relative to cells that primarily express IRAK4 [50]. Drugs targeting IRAK-4 have been mentioned in Table 1.

#### Role of inflammasomes in MDS

Inflammasomes are intracellular, cytoplasmic, multiprotein, high molecular weight immune complexes that play an important role in the host defense against pathogens [61]. The receptor proteins that can assemble to form inflammasomes include *n*ucleotide-binding domain leucine-rich repeat (LRR)-containing protein (NLR) family members NLRP1, NLRP2, NLRP3, NLRP6, NLRP7, NLRC4 (*N*od-*l*ike *r*eceptor *C*ARD domain-containing 4) and the HIN-200 family member Aim2 (*a*bsent*i*n*m*elanoma*2*). Inflammasomes sense intracellular DAMPs and PAMPs leading to the activation of inflammatory caspases such as caspase 1 [62, 63]). NLRP3 polymerizes apoptosis-associated speck–like protein (ASC) which contains a pyrin domain (PYD) and a caspase recruitment domain (CARD). ASC binds to inflammasomes through PYD and recruits procaspase 1 via CARD [61, 62]. Proximity-dependent auto-activation of pro-caspase-1 leads to the formation of active caspase 1, which in turn activates pro-IL-18 and pro-IL-1β into their mature forms. Caspase 1 mediates pore forming protein gasdermin-D (GSDMD) dependent pyroptotic lytic cell death, causing the release of mature IL-1β and IL-18, DAMPs, ATP, DNA, and even inflammasomes themselves which propagate inflammation [61, 62]. (Fig. 2).

#### Pathogenesis of inflammasomes in MDS

There is increasing evidence supporting the role of inflammasomes in different phases of MDS pathogenesis including pyroptosis of HSPCs causing cytopenia, macrocytosis, ineffective hematopoiesis, and the β-catenin induced proliferation of cancer cells [64]. Mononuclear cells from the bone marrow of MDS patients revealed profound upregulation of caspase-1 (~ 209-fold) and NLRP3 (~ 48-fold) with no difference in caspase-3 expression. Pyroptosis execution (active caspase-1+/active caspase-3+/annexin-V- cells) was a predominant form of cell death when compared to apoptosis (active caspase-3/7+/active caspase-1−/annexin-V+) [62, 64]. Mutations in MDS related genes and S100 A8/A9 can cause activation of NLRP3 inflammasome promoting pyroptosis. It is commonly seen in 5q deletion MDS, causing the activation of p53-S100A8/9-TLR4 axis [62]. Basiorka et al. (2016) reported that somatic gene mutations, irrespective of functional class, can activate the NLRP3-pyroptosis axis, and the extent of pyroptosis is directly proportional to the burden and complexity of somatic gene mutations [65]. RNA splicing gene (*U2AF1, SF3B1*, and *SRSF2*) and epigenetic regulatory gene (*ASXL1 and TET2*) mutations in HSPCs of mouse models induced pyroptosis which was suppressed by NLRP3 inhibition [64, 65].

#### Drugs targeting inflammasomes

NLRP3 activation and apoptosis play a crucial role in the pathogenesis of MDS. Although specific NLRP3 targeting agents are still in preclinical development, they are showing promising results with a good safety profile in mice. Some direct NLRP3 inhibitors include 3,4-methylenedioxy-β-nitrostyrene (MNS) and CY-09, an analog of CFTR inhibitor-172 (C172) which inhibits the CFTR channel, specifically targets ATP binding of NLRP3, but these inhibitors are still in the stage of in vitro and in vivo studies [64, 66–68].

IL-1β antagonists such as anakinra, canakinumab, and rilonacept have been approved for the treatment of several other autoinflammatory conditions. It has been reported that prolonged IL-1β elevation can cause chronic stimulation of NF-κB and MAPK signaling. Hence, IL-1β can be a potential effective target in MDS [64, 66]. However, further investigations have to be done to determine the role of IL-1β antagonists in MDS. Currently, canakinumab (an IL-1β–neutralizing monoclonal antibody) is being studied in early phase clinical trials (NCT04810611, NCT04239157) [66]. Ibrutinib, a BTK inhibitor that prevents the formation of ASC specks and Caspase 1 activation is used in combination with lenalidomide and 5′-Azacytidine is under phase 1 clinical trials for MDS (NCT03359460 and NCT02553941) [66, 69]. Caspase 1 inhibitors, such as VX-765, and soluble analogs of Parthenolide (an anti-inflammatory sesquiterpene lactone compound) can play a therapeutic role in MDS. However, further research needs to be done on their efficacy. GSDMD has also been identified as a potential therapeutic target, and it could become a crucial protein to prevent pyroptosis in the future [66, 69] (Table 1).

### Role of transforming growth factor beta (TGF-β) in MDS

Transforming growth factor beta (TGF-β) is a large superfamily of growth factors that includes activins and bone morphogenetic proteins. They play a role in maintaining the proliferation and differentiation of the HSC [70]. The TGF-β family plays an important role in embryogenesis, as alterations in the pathway are associated with developmental abnormalities, autoimmune disorders, and carcinogenesis [71]. TGF-β ligands bind to cell surface receptors which are classified as type I and type II. The majority of the pathway signaling occurs via a type I receptor activin like kinase 5 (ALK 5) or TβRII [72] (Fig. 1). Upon activation, these receptors fire the downstream SMAD signaling circuit, which functions as the major signaling pathway of the TGF-β pathway [72]. The SMAD family includes eight subtypes (SMAD 1–8) with varying functions that can be either activating (SMAD 2/3) or inhibitory (SMAD 6/7) in nature [73]. TGF-β functions as a major inhibitor of the HSCs via the upregulation of cyclin-dependent kinase inhibitors, such as p15Ink4b, p21Cip1, and p27Kip1 and, in turn, maintains the quiescent state of HSC and prevents its loss [74]. SMAD signaling also depends on other coregulatory signals in the bone marrow niche, such as the nuclear protein Transcriptional Intermediary Factor 1γ (TIF1γ) and SMAD 4. In vivo models have shown that SMAD 4 inhibits the SMAD 2/3 complex, resulting in the suppression of HSC transcription, but TIF1γ competes with SMAD 4 for binding to the phosphorylated SMAD 2/3 complex and thereby promotes erythroid differentiation [75]. The pathway is also regulated by inhibitors such as SMAD7 and Ski protein, corepressor for SMAD-4 [76, 77].

The inhibitory TGF-β pathway is normally tightly regulated, but chronic TGF- β signaling is noted early in the course of MDS. Alterations in SMAD 7 and SKI proteins result in the sustained activation of pathways and lead to ineffective erythropoiesis [78]. Human CD 34 + cell lines have also shown elevated levels of microRNA 21 (miR-21) that cause decreased levels of SMAD 7, culminating in the decreased formation of erythroid colonies as noted in a comparison of MDS patients to healthy controls [78]. Similarly, SMAD 2 is upregulated in MDS, and the in vivo pharmacologic inhibition of ALK 5 or the chemical inhibition of miR-21 increased hemoglobin levels in a mouse model [79]. Murine models have shown that the trapping of Activin and GDF11 with RAP-536 inhibits the activation of SMAD 2/3 proteins and helps relieve the ineffective erythropoiesis seen in MDS [80]. In the MEDALIST trial, Luspatercept significantly reduced transfusion burden in low-risk MDS patients with ring sideroblasts refractory or intolerant or unlikely to respond with erythropoiesis-stimulating agents [81]. Luspatercept is a recombinant fusion protein of the extracellular domain of human Activin receptor 2B and IgG1 Fc domain which binds TGF β ligands to reduce SMAD2 and SMAD3 signaling resulting in erythroid differentiation [81].

### Role of S100A8/A9 in MDS

S100 proteins are calcium-binding cytosolic proteins labeled due to their solubility in 100% saturated ammonium sulfate [82, 83]. Members of the S100 family perform a wide range of functions at both the extracellular and intracellular levels, including cell proliferation, differentiation, apoptosis, migration, inflammation, calcium homeostasis, and enzyme regulation [84]. S100A8 also called the migration inhibitory factor-related protein-8 (MRP-8)/calgranulin A, and S100A9, also called the migration inhibitory factor-related protein-14 (MRP-14)/calgranulin B, act as alarmins or DAMPs [82, 84]. They are released from immunocytes and circulate in the plasma as homodimers or as S100A8/A9 heterodimers also called calprotectin. The levels of S100A8 and S100A9 are high in autoimmune conditions, inflammation, and cancer [82, 84]. They are predominantly expressed in neutrophils, monocytes, and dendritic cells. S100A8 and S100A9 constitute around 45% and 1% of the cytosolic proteins in neutrophils and monocytes, respectively. However, upon activation by cell damage/stress, they are also expressed in various other cells such as mature macrophages, fibroblasts, erythroblasts, and vascular endothelial cells [85].

### Mechanism of signaling and the role of S100 proteins in MDS

When S100 proteins are released from immunocytes due to cell damage/stress, they act as danger signals and play a significant role in inflammatory response facilitating apoptosis, autophagy, chemotaxis, invasion, and differentiation [85]. S100A8/A9 proteins bind to TLRs, CD33 and the receptor for advanced glycation end products (RAGE), thereby mediating several downstream effects such as the generation of inflammatory cytokines such as IL-6, TNF alpha, and IL-1 Beta, which in turn stimulate NF-κB activation leading to activation of inflammasome and osmotic lysis [64, 85, 86] (Fig. 1), (Fig. 2).

In an in vivo study conducted by Cheng et al. (2013), it was reported that the S100A9/CD3 pathway caused the stimulation of polyclonal MDSCs (CD33+/Lin−/HLA − DR−) in the bone marrow and was responsible for hematopoietic senescence in MDS. CD3 is a member of the sialic acid-binding Ig-like superfamily of lectins (Siglec) and possesses an immunoreceptor tyrosine-based inhibition motif (ITIM) which is associated with immunosuppression [64, 87]. When S100A9 binds to CD3, this ligand-receptor complex causes ITIM mediated production of immunosuppressive cytokines like IL-10 and TGF-β, which repress erythropoiesis. Data also shows the restoration of hematopoiesis by breaking the S100A9/CD3/TLR4 circuit. Hence, S100A9 can be an initiating factor for the sequence of inflammatory responses leading to defective erythropoiesis in MDS [64, 87]. Furthermore, a study was conducted by Schneider and his colleagues to investigate the 5q deletion phenotype of MDS. This study particularly focused on the molecular consequences of ribosomal protein small subunit 14 (Rps14) haploinsufficiency that happens with 5q deletion using a conditional-knockout mouse model [88]. Data showed that the Rps14 haploinsufficient bone marrow cells caused stress leading to the overexpression of S100A8/9 proteins in monocytes, macrophages, and late-stage erythroblasts. This process in turn induced the expression of P53, mediating erythroid differentiation defects. A pharmacological intervention targeting the inactivation of S100A8/A9 could improve erythropoiesis in 5qdel MDS [88].

### Role of interleukin-8 in MDS

Interleukin-8 (IL-8) is a member of the CXC chemokine subfamily, produced by blood cells, and it acts on neutrophils, attracting them to the site of inflammation [89]. IL-8 activates multiple intracellular signaling pathways in the neutrophils, allowing their pathophysiological role such as neutrophil degranulation and chemotaxis [90]. Elevated levels of IL-8 and its receptors have been reported in cancer cells, endothelial cells, infiltrating neutrophils, and tumor-associated macrophages, thereby activating multiple downstream signaling pathways, such as serine/threonine kinases which increase MAPK signaling and the activation of IL-8 in ovarian and lung cancer cell lines mediates angiogenesis, cell motility, and invasion [91]. Due to the hypoxia in the bone marrow microenvironment, high levels of expression of IL-8 are noticed in AML cell lines and are associated with a poor prognosis [92]. The role of IL-8 in MDS is evolving, and the overexpression of the IL-8 receptor CXCR2 was observed in AML/MDS CD34 + stem cell lines as compared to healthy CD34 + controls. CXCR2 inhibition in these cell lines led to selective inhibition of immature hematopoietic stem cell lines, sparing the control cell lines and providing a potential therapeutic target [93]. Drugs targeting IL-8 and IL-8 receptors are mentioned in Table 1.

### Low-risk and high-risk MDS

Innate inflammatory signaling also influences the differentiation and expansion of hematopoietic stem cells (HSCs) and can be differentially activated in low-risk and high-risk MDS phenotypes [94, 95]. Apoptotic pathways predominate in bone marrow in low-risk MDS phenotypes while high-risk MDS clones are able to evade the immune system aiding its progression to AML [96]. The most common cytogenetic abnormality in lower risk MDS, del(5q), is associated with dysregulated innate immune pathways as many genes involved in innate immune signaling, such as *DIAPH1, TIFAB, MiR-146a*, etc. are in close proximity to the 5q region [97]. In higher risk MDS, T regulatory cells predominate leading to suppressed immune surveillance and hence disease progression [98]. Several studies have also reported that autoimmune diseases cluster in high-risk MDS phenotypes suggesting a common inflammatory link in the pathogenesis of these diseases [99–101]. VEXAS (Vacuoles, E1 enzyme, X linked, autoinflammatory, somatic) syndrome is an example dysregulated innate pathway that links autoimmune disease with MDS. This syndrome is due to somatic mutation of Ubiquitinin-like modifier activating enzyme 1 gene (*UBA1*) located on X chromosome leading to upregulation of unfolded protein accumulation and increased stress in myeloid cells causing bone marrow failure [102].

## Conclusion

Understanding the role of innate immune dysregulation has improved over time. There have been a number of preclinical and early phase clinical studies which have investigated novel therapies targeting these pathways. Unfortunately, despite their impressive progress, effective therapeutic options remain very limited. Additionally, there are still several important, lingering questions dealing with the role of innate immunity in pathogenesis of MDS such as: (1) Which immune pathway is the best to target in treating MDS? (2) Which age-related and microenvironmental changes favor the development of the MDS phenotype? Even though promising early activity is being seen with IRAK and TGF inhibitors, we hope future trials will be able to answer these questions comprehensively. Continued endeavors to gain a deeper understanding of innate immunity’s role, discover effective target molecules, and translate them into preclinical and early phase clinical trials will hopefully bring promising therapies in the future.

## Data Availability

All data generated during this study are included in this article.

## Abbreviations

AML: Acute myeloid leukemia
BCL2: B-cell lymphoma 2
BTK: Bruton tyrosine kinase
CpG: CpG oligodeoxynucleotide
CFU: Colony-forming unit
CLL: Chronic lymphocytic leukemia
DAMPs: Damage associated molecular patterns
del(5q): deletion of the long arm of chromosome 5
EZH2: Enhancer of zeste homolog 2
GCSF: Granulocyte colony-stimulating factor
GMCSF: Granulocyte-macrophage colony-stimulating factor
HSC: Hematopoietic stem cell
HSPCs: Hematopoietic stem/progenitor cells
IFN: Interferon
IFN-γ: Interferon-gamma
IL-1: Interleukin-1
IL-6: Interleukin-6
IL-8: Interleukin-8
IPSS-R: International Prognostic Scoring System-Revised
IRAK: Interleukin-1 receptor-associated kinase
IRAK1: Interleukin-1 receptor-associated kinase1
IRAK4: Interleukin-1 receptor-associated kinase4
IRF-3: Interferon Regulatory Factor-3
ISREs: IFN stimulated response elements
JNK: c-Jun N-terminal kinase
kDa: KiloDaltons
LPS: Lipopolysaccharide
MAP: Mitogen-activated protein
MAPK: Mitogen-activated protein kinase
MDSC: Myeloid-derived suppressor cell
MDS: Myelodysplastic syndrome
miR: MicroRNA
miRNAs: MicroRNAs
MKP-1: Mitogen-activated protein kinase-1
MyD88: Myeloid differentiation primary response 88
NEMO: NF-kB essential modulator
NF-κB: Nuclear factor-kappa B
NLRP3: NOD-like receptor family, pyrin domain-containing 3
PAMP: Pathogen-associated molecular pattern
PAMPs: Pathogen-associated molecular patterns
PRRs: Pattern recognition receptors
RIP: Receptor-interacting protein
SF3B1: Splicing factor 3B subunit 1
siRNA: Small interfering RNA
SRSF2: Serine- and arginine-rich splicing factor 2
STAG2: Stromal antigen 2
STAT3: Signal transducer and activator of transcription 3
TBK 1: TANK-binding Kinase 1
TH1: T helper 1
TLR: Toll-like receptor
TLR4: Toll-like receptor4
TLRs: Toll-like receptors
TIR: Toll/interleukin-1 receptor
TIRAP: Toll/interleukin-1 receptor (TIR) domain-containing adapter protein
TRAF: TNF receptor associated factor
TRAF6: TNF receptor-associated factor6
TIFAB: TRAF-interacting protein with a forkhead-associated domain B
TRAM: TRIF-related adapter molecule
TRIF: TIR domain-containing adapter-inducing interferon-beta
U2AF1: U2 small nuclear RNA auxiliary factor 1
